# Symptoms, unbearability and the nature of suffering in terminal cancer patients dying at home: a prospective primary care study

**DOI:** 10.1186/1471-2296-14-201

**Published:** 2013-12-28

**Authors:** Cees DM Ruijs, Ad JFM Kerkhof, Gerrit van der Wal, Bregje D Onwuteaka-Philipsen

**Affiliations:** 1 Department of Public and Occupational Health, Expertise Center for Palliative Care, VU University Medical Center, EMGO+Institute, van der Boechorststraat 7, 1081 BT Amsterdam, The Netherlands; 2 Department of Clinical Psychology, VU University, EMGO+Institute, van der Boechorststraat 1, 1081 BT Amsterdam, The Netherlands; 3 Primary Care Center De Greev, Grevelingenstraat 10, 3522 PR Utrecht, The Netherlands

## Abstract

**Background:**

Primary care physicians provide palliative home care. In cancer patients dying at home in the Netherlands (45% of all cancer patients) euthanasia in about one out of every seven patients indicates unbearable suffering. Symptom prevalence, relationship between intensity of symptoms and unbearable suffering, evolvement of symptoms and unbearability over time and quality of unbearable suffering were studied in end-of-life cancer patients in primary care.

**Methods:**

44 general practitioners during three years recruited cancer patients estimated to die within six months. Every two months patients quantified intensity as well as unbearability of 69 symptoms with the State-of-Suffering-V (SOS-V). Also overall unbearable suffering was quantified. The five-point rating scale ranged from 1 (not at all) to 5 (hardly can be worse). For symptoms assessed to be unbearable the nature of the suffering was additionally investigated with open-ended questions. The final interviews were analyzed; for longitudinal evolvement also the pre-final interviews were analyzed. Symptom intensity scores 4 and 5 were defined to indicate high intensity. Symptom unbearability scores 4 and 5 were defined to indicate unbearable suffering. Two raters categorized the qualitative descriptions of unbearable suffering.

**Results:**

Out of 148 requested patients 51% participated; 64 patients were followed up until death. The SOS-V was administered at least once in 60 patients (on average 30 days before death) and at least twice in 33 patients. Weakness was the most frequent unbearable symptom (57%). Pain was unbearable in 25%. Pain, loss of control over one’s life and fear of future suffering frequently were unbearable (89-92%) when symptom intensity was high. Loss of control over one’s life, vomiting and not being able to do important things frequently were unbearable (52-80%) when symptom intensity was low. Unbearable weakness significantly increased between pre-final and final interview. Physical suffering, loss of meaning, loss of autonomy, experiencing to be a burden, fear of future suffering and worrying more frequently occurred in patients suffering unbearably overall.

**Conclusions:**

Weakness was the most prevalent unbearable symptom in an end-of-life primary care cancer population. Physical suffering, loss of meaning and loss of autonomy more frequently occurred in patients who suffered unbearably overall.

## Background

Many cancer patients in the end-stage of their disease prefer to die at home [1,2]. In the Netherlands 45% of all cancer patients die at home [3]. Palliative care for these patients is provided by primary care physicians. Of the remaining cancer deaths 31% occur in hospitals, 19% in care homes and 4% in hospices. Characteristics of primary palliative care include selection of patients with a preference to die at home, palliative care provided by a physician trained in general medicine, strong relationships between physicians and patients, care provided at home and dependent upon the possibilities of care at home, support by a home team, support by a specialized palliative care service and negative selection of cancer related emergencies [4-7]. The low prevalence of patients dying from cancer at home may interfere with effectively building up experience in palliative cancer care [8]. Primary care in the Netherlands is provided by nearly 9.000 general practitioners (GPs), 57% of whom work part time [9]. A full time GP on average is responsible for palliative care for a cancer patient nearly three times a year [10].

Suffering in advanced cancer patients [11] may result in a desire for hastened death, or even a request for euthanasia or physician-assisted suicide [EPAS] [12-16]. In a few countries and states, including the Netherlands, EPAS has been legalized [17,18]. EPAS in legalized settings in majority is performed in primary care [18,19]. In relation to continuing societal and political debate about end-of-life decision making countries and states may face reevaluation of the legal position of EPAS [20,21]. The legalization of EPAS in the Netherlands requires presence of unbearable suffering, which needs to be assessed present by a physician who considers to perform EPAS in reaction to an explicit request for EPAS. Unbearable and hopeless suffering, no reasonable acceptable treatment alternative, a voluntary and well-considered request and consultation of an independent physician are among the compulsory criteria for EPAS [17]. EPAS is not permitted if the suffering is not assessed to be unbearable.

In professional literature the supposition holds that the proportion of patients who seriously consider EPAS is small [15,22]. Research seldom addresses unbearable suffering, nor EPAS, through prospective patient directed research. In the Netherlands in 2010 42.600 patients died as a consequence of cancer [23], of whom upon estimation ~ 19.000 (45%) [3] died at home. A total of ~3550 EPAS (88% of in total 4050 annual cases of EPAS) was performed in primary care [19] of which in ~ 2800 cases (cancer is the diagnosis in 79% of cases of EPAS) the diagnosis was cancer. Thus, of end-of-life cancer patients cared for in primary care around one in seven died as a consequence of EPAS. This proportion is considerable and underscores the importance for a better understanding of the nature of unbearable suffering in various settings of care, which may contribute to competently providing care for patients who experience unbearable suffering.

Unbearable suffering seldom is investigated through prospective, patient directed research. Intensity of symptoms is considered to largely determine the suffering of incurable cancer patients [24]. However, a symptom that is unbearable for one person may be bearable for another. A prospective study in end-of-life cancer patients cared for in primary care was performed, addressing the following questions: Which are the most prevalent symptoms? What is the relationship between intensity of symptoms and unbearable suffering? How do symptoms and unbearable suffering evolve over time? Which qualitative characteristics of unbearable symptoms determine the nature of unbearable suffering?

## Methods

### Design

The study was conducted in Utrecht, a city with a population of 235 000 people and 105 GPs. Eligible were terminal cancer patients expected to die within six months and expected to live at home (most of the time) until death cared for by a GP as the primary responsible physician. GPs requested eligible patients to participate. GPs estimated survival based upon signs of clinical deterioration of the patient.

Forty-four GPs, 59% of whom worked part time, representing 42% of the GPs in the city, with practice locations dispersed throughout the city, requested eligible patients to participate. A researcher visited the consenting patients within a week and administered the baseline interview. Follow-up interviews were every two months, or sooner if the condition of a patient deteriorated. All interviews were at the patients’ residence. The recruitment process was organized by a study coordinator. GPs were personally contacted every two months to identify newly eligible patients. Baseline characteristics of all eligible patients were registered. The interviewers were a physiotherapist (the study coordinator) and a GP (CDMR), both trained in interview techniques. The study protocol was approved by the Medical Ethics Committee at the VU University Medical Center. The recruitment process is described in detail elsewhere [25].

### Study population

Patient recruitment was from May 2003 until May 2006, and follow-up continued until May 2007. Seventy six out of 148 invited patients (51%) entered the interview study. The attrition rate was 8%, caused by patients who stopped participating after one or more interviews and at the end of follow-up 8% of the patients were alive, leaving 64 patients with follow up until death. Main reasons for declining were unexpected rapid physical deterioration (N = 27), considering participation too burdensome (N = 20) and disliking to talk (N = 15). Another 110 patients were not invited by the GP, because unexpected rapid disease progression resulted in a physical condition too debilitated to sustain an interview. Age, gender and type of cancer did not differ between the patients in and out of the interview sample. The prevalence of seriously depressed mood according to the GP was significantly lower in the interview sample: 5% versus 23% in the sample which declined participation and 14% in the sample which was not asked to participate because of a too debilitated physical condition [26]. The prevalence of depression was investigated, the results of which have been published separately; one patient suffered from a definite major depression [26].

### Measurement instrument: State-of-Suffering-V

Quality of life instruments tend to focus on the intensity of symptoms. Health-related quality of life instruments have limitations in the context of research in dying patients [27]. No instrument existed to assess unbearable suffering. The State-of-Suffering-V (SOS-V) was developed [28], an instrument directed at assessing the experience of unbearable suffering of the whole person [11]. The SOS-V (Additional file 1) is a patient-driven instrument, which provides quantitative assessment of intensity and unbearability of physical, psychological, social and existential symptoms which may cause suffering. “Symptoms” refers to medical symptoms and circumstances as well as psychological, social and existential aspects which may cause suffering. This extended interpretation of symptoms is not uncommon in psychological research. The SOS-V addresses 69 symptoms in a framework of five functional domains: (I) medical signs and symptoms; (II) loss of function; (III) personal aspects; (IV) environment (psychosocial support, provided care) and (V) nature and prognosis of the disease [28].

Physical symptoms largely are present in the domain of medical signs and symptoms. The nature of suffering caused by a certain symptom cannot be predicted and therefore an a-priori distinction of suffering into physical, psychosocial, or existential dimensions cannot be made.

The SOS-V is a structured instrument in which the patient assesses intensity (or extent) and unbearability per symptom. A uniform 5-point scoring scale is employed, supported by a description: 1-not at all; 2-slightly; 3-moderately; 4-seriously; 5-very seriously, hardly can be worse. Whenever a patient rates a 4 or 5 for unbearability of a symptom the interviewer through open-ended questions shortly further explores the experience and immediately writes down exact phrases of the answers. After rating the 69 symptoms the interviewer asks whether there are missing aspects which cause suffering, and rates these accordingly if present. Thereafter the patient is asked to rate overall unbearable suffering, considering all the present symptoms (same rating scale). Semi-structured administration of the SOS-V is permitted in the sense that the interviewer may follow a spontaneous other sequence of symptoms which a patients provides, as long as all symptoms are evaluated. The reference period in the study was the last two days. Limited field testing was performed. The development of the instrument, including analysis of validity, is described elsewhere [28]. Administration of the quantitative questions of the SOS-V most times was possible within 15 to 20 minutes [10,28].

### Analysis of relationship between intensity of symptoms and unbearable suffering

The final SOS-V interviews before death in 60 patients were analyzed to investigate the relationship between intensity of a symptom and experienced unbearable suffering caused by that symptom. Four patients could not be evaluated because the interviewer considered the interview to burdensome and abandoned the interview.

Intensity of symptoms and unbearable suffering per symptom were analyzed dichotomously. A symptom with intensity rated 2 (slightly) or 3 (moderately) was defined to be of low intensity, while a symptom rated 4 (seriously) or 5 (hardly can be worse) was defined to be of high intensity. Symptoms in which unbearability was rated 1 (not at all), 2 (slightly) or 3 (moderately) were defined to be bearable, while symptoms rated 4 (seriously) or 5 (hardly can be worse) were defined to be unbearable. Overall unbearable suffering was defined accordingly. Presence of symptoms, intensity of symptoms and unbearability per symptom are presented as proportions of interviewed patients. Sample size (many clinically relevant symptoms were infrequently present) interfered with worthwhile statistical analysis. The relationship between intensity of symptoms and unbearable suffering is evaluated for the symptoms which (arbitrarily) were unbearable in at least 15% (n = 9) of the patients.

### Analysis of longitudinal data

In 33 patients the SOS-V was administered at least two times. Intensity of symptoms, consequent unbearable suffering, and overall unbearable suffering, were analyzed for the symptoms in which unbearable suffering occurred most frequently (symptom unbearable in at least 15% of patients). For dichotomous analysis the same cut-off scores were used as described above. Confidence intervals for paired data per patient were calculated for statistical analysis.

### Analysis of subjective descriptions of unbearable suffering

The answers of the patients to open questioning of why the suffering caused by a symptom was unbearable were analyzed. Patients in whom overall suffering was bearable were compared with patients in whom overall suffering was unbearable. A schedule was made of senses of suffering [29] considered important in end-of-life cancer patients (Table 1) [11,24,29-35]. Common emotional symptoms of psychological distress of individuals as they approach the terminal phase of illness [36,37] were additionally analyzed together. Two raters, a GP (CDMR) and an external clinical psychologist, rated all the qualitative answers. It was only permitted to give one rating per unbearable answer: the best fitting match. The raters started with independently rating the answers of five patients, after which the answers were compared. Fine tuning of the rating process was applied based upon exchange of arguments, after which the answers of the remaining patients were independently rated. Then the raters compared all differing ratings and applied one rating if consensus could be reached. Only equal ratings were used to compare patients in whom overall suffering was bearable with patients in whom overall suffering was unbearable. Consensus between the raters occurred in 86% of the analyzed descriptions of unbearable suffering. T-tests for mean prevalence were used for statistical analysis.

**Table 1:** Categorization of qualitative data

**Category of suffering**	**Indications for assigning category**
Physical	Medical morbidity, the physical symptom itself, physical symptoms which result in physical experienced suffering
Loss of meaning	Loss of: identity, capacity of self-fulfillment, communication, social role, social interaction, intimacy
Loss of autonomy	Suffering acknowledged to be caused by: loss of autonomous functioning or occurrence of dependency (presence itself of loss of autonomy or of dependency is not sufficient to assign the category)
Loss of dignity	Socially embarrassing symptoms, shame, body image concerns, not being taken seriously, worthlessness
Loss of sexual role	Loss of capability of sexual functioning; loss of sexual role
Fear of future suffering	Fear caused by awareness of potential suffering related to progress of disease
Anxiety	Anxiety
Death anxiety	Anxiety related to awareness of the process of dying and what will go along with that, and anxiety related to the actual dying process
Depressiveness	Suffering caused by the presence of depressive thoughts
Worrying	Negative thoughts which cannot be turned off
Feeling tensed	Feeling tensed in mind or body
Hopelessness	Loss of possibility of meaning
Pointlessness	Total loss of meaning; nothing left

In the ratings which remained without consensus it was analyzed what caused the difference in rating. Based upon an analytical process two possibilities were identified. The first was different opinion about interpretation of the answer. The second was that the answer contained varied information, which made various ratings applicable. Persistent difference in rating in 9% was attributable to different interpretation of the answers of the patients. In 5% difference in rating occurred because the answers contained information which was applicable to more than one rating.

## Results

### Study sample

The studied sample consisted of 60 patients; 46 patients died within six months. The final interview on average was 30 days before death (SD 17 days); in 23% the interview was within 2 weeks prior to death. The average age of the patients was 70 years (range 38–86), 52% were female, 60% were educated beyond elementary school, 63% were living alone, 77% had children and 62% were religious (protestant or catholic). The most prevalent malignancies were lung cancer (27%) and gastro-intestinal cancer (25%).

### Symptom prevalence and unbearable suffering

Symptom prevalence was highest in the domains of medical symptoms, loss of function and personal aspects (Tables 2 and 3). Weakness was the most prevalent symptom in the domain of medical symptoms (93%), and was unbearable in 57% of the patients. Other prevalent symptoms were tiredness, general discomfort, changed appearance and pain (72-87%). Pain was unbearable in 25%. Most prevalent in the functional domain were impaired routine daily activities and impaired leisure activities (just above 80%), which were unbearable in half of the patients. Most prevalent in the domain of personal aspects was feeling dependent upon others (80%), which was unbearable in 45% of patients. Other prevalent symptoms were not being able to do important things and trouble accepting the present situation (around 60%). Loss of control over one’s life was sensed in 30% of patients and was unbearable in 27%. In the domain of environment the feeling that relatives considered the suffering too severe occurred in 33%. In the domain of nature and prognosis of disease fear of future suffering occurred in 40%.

**Table 2:** Relationship between symptom intensity and unbearability for symptoms in the domain of: medical signs and symptoms (n = 60)

	Symptom present % *(n)	Symptom high intensity % *(n)	High intensity and unbearable % *(n)	**Percentage unbearable when high intensity %**	Symptom low intensity % *(n)	**Low intensity and unbearable %(n)**	**Percentage unbearable when low intensity %**	Symptom unbearable % *(n)
***Domain I: Medical symptoms***								
Weakness	93 (56)	51 (31)	43 (26)	84	42 (25)	14 (8)	32	57 (34)
Tiredness	87 (52)	48 (29)	28 (17)	59	39 (23)	7 (4)	17	35 (21)
General discomfort	80 (48)	27 (16)	17 (10)	63	53 (32)	20 (12)	38	37 (22)
Changed appearance	78 (47)	40 (24)	20 (12)	50	38 (23)	2 (1)	4	22 (13)
Pain	72 (43)	20 (12)	18 (11)	92	52 (31)	7 (4)	13	25 (15)
Loss of appetite	62 (37)	24 (14)	10 (6)	43	38 (23)	15 (9)	39	25 (15)
Shortness of breath	59 (35)	17 (10)	14 (8)	80	42 (25)	5 (3)	12	19 (11)
Impaired co-ordination	57 (34)	17 (10)	13 (8)	80	40 (24)	5 (3)	13	18 (11)
Not sleeping well	47 (28)	12 (7)	10 (6)	86	35 (21)	15 (9)	43	25 (15)
Thirst	45 (27)	18 (11)	10 (6)	55	27 (16)	2 (1)	6	12 (7)
Feeling tensed	44 (26)	12 (7)	10 (6)	86	32 (19)	2 (1)	5	12 (7)
Memory loss	43 (26)	12 (7)	7 (4)	57	32 (19)	8 (5)	26	15 (9)
Impaired sight	42 (25)	5 (3)	3 (2)	67	37 (22)	4 (2)	9	7 (4)
Impaired mental clarity	42 (25)	10 (6)	5 (3)	50	32 (19)	7 (4)	21	12 (7)
Loss of concentration	40 (24)	13 (8)	12 (7)	88	27 (16)	5 (3)	19	17 (10)
Coughing	38 (23)	2 (1)	2 (1)	100	36 (22)	3 (2)	9	5 (3)
Swallow food impaired	35 (21)	7 (4)	7 (4)	100	28 (17)	5 (3)	18	12 (7)
Smelling unpleasant	35 (21)	8 (5)	8 (5)	100	27 (16)	3 (3)	19	13 (8)
Feeling depressed	34 (20)	3 (2)	2 (1)	50	31 (18)	10 (6)	33	12 (7)
Impaired hearing	33 (20)	8 (5)	7 (4)	80	25 (15)	8 (4)	27	13 (8)
Incomprehensible speech	32 (19)	14 (8)	12 (7)	88	18 (11)	3 (2)	18	15 (9)
Itch	32 (19)	8 (5)	5 (3)	60	24 (14)	2 (1)	7	7 (4)
Constipation	30 (18)	8 (5)	8 (5)	100	22 (13)	4 (2)	15	12 (7)
Nausea	28 (17)	12 (7)	8 (5)	71	16 (10)	5 (3)	30	13 (8)
Dizziness	27 (16)	5 (3)	5 (3)	100	22 (13)	7 (4)	31	12 (7)
Vomiting	27 (16)	9 (5)	7 (4)	80	18 (11)	13 (8)	73	20 (12)
Feeling anxious	27 (16)	5 (3)	3 (2)	67	22 (13)	4 (2)	15	7 (4)
Swallowing fluid impaired	23 (14)	5 (3)	5 (3)	100	18 (11)	2 (1)	9	7 (4)
Hiccups	22 (13)	7 (4)	5 (3)	75	15 (9)	5 (3)	33	10 (6)
Intestinal cramps	22 (13)	5 (3)	3 (2)	67	17 (10)	5 (3)	30	8 (5)
Diarrhea	20 (12)	3 (2)	3 (2)	100	17 (10)	4 (2)	20	7 (4)
Incontinence of urine	10 (6)	0 (0)	0 (0)	0	10 (6)	0 (0)	0	0 (0)
Incontinence of feces	8 (5)	2 (1)	2 (1)	100	6 (4)	5 (3)	75	7 (4)
Pressure ulcers	8 (5)	2 (1)	2 (1)	100	6 (4)	1 (1)	25	3 (2)
Impaired comprehension of speech	7 (4)	2 (1)	2 (1)	100	5 (3)	1 (1)	33	3 (2)
Paralyzed limbs	5 (3)	2 (1)	2 (1)	100	3 (2)	0 (0)	0	2 (1)
Skin metastasis	3 (2)	3 (2)	2 (1)	50	0 (0)	0 (0)	0	2 (1)

Rounded percentages and absolute numbers; between 0 to 1 missing observations per symptom.

Scoring: 1-not at all; 2-slightly; 3-moderately; 4-seriously; 5-very seriously, hardly can be worse.

Symptom present: intensity scores 2–5.

Low intensity: scores 1–3; High intensity: scores 4–5; Bearable suffering: scores 1–3; Unbearable suffering: scores 4–5.

*: percentage of interviewed patients followed up until death.

n: number of patients.

**Table 3:** Relationship between symptom intensity and unbearability in the domains of loss of function, personal aspects, environment, nature and prognosis of disease (n = 60)

	Symptom present %*(n)	Symptom high intensity %* (n)	High intensity and unbearable %*(n)	**Percentage unbearable when high intensity %**	symptom low intensity %*(n)	Low intensity and unbearable %*(n)	**Percentage unbearable when low intensity %**	Symptom unbearable %*(n)
***Domain II: Loss of function***								
Impaired routine daily activities	83 (50)	58 (35)	48 (29)	83	25 (15)	7 (4)	27	55 (33)
Impaired leisure activities	82 (49)	60 (36)	48 (29)	81	22 (13)	2 (1)	8	50 (30)
Help needed with housekeeping	71 (42)	54 (32)	39 (23)	72	17 (10)	3 (2)	20	42 (25)
Help needed with self-care	60 (36)	27 (16)	15 (9)	56	33 (20)	7 (4)	20	22 (13)
Bedridden	56 (33)	32 (19)	24 (14)	74	24 (14)	8 (5)	36	32 (19)
Impaired working capacity	17 (10)	17 (10)	12 (7)	70	0 (0)	0 (0)	0	12 (7)
Impaired sexual functioning	14 (8)	12 (7)	5 (3)	43	2 (1)	0 (0)	0	5 (3)
***Domain III: Personal aspects***								
Feeling dependent on others	80 (48)	50 (30)	37 (22)	73	30 (18)	8 (5)	28	45 (27)
Not able to do things you consider important	63 (36)	26 (15)	23 (13)	87	37 (21)	19 (11)	52	42 (24)
Trouble accepting the present situation	60 (36)	30 (18)	25 (15)	83	30 (18)	8 (5)	28	33 (20)
Feeling a nuisance to others	38 (23)	5 (3)	3 (2)	67	33 (20)	10 (6)	30	13 (8)
Negative thoughts or worrying	32 (19)	12 (7)	8 (5)	71	20 (12)	7 (4)	33	15 (9)
Loss of control over your own life	30 (18)	22 (13)	20 (12)	92	8 (5)	7 (4)	80	27 (16)
Feeling not any longer being the same person	28 (17)	8 (5)	8 (5)	100	20 (12)	2 (1)	8	10 (6)
Hopelessness	28 (17)	7 (4)	7 (4)	100	21 (13)	6 (4)	31	13 (8)
Experienced little happiness with family/friends	22 (13)	5 (3)	0 (0)	0	17 (10)	8 (5)	50	8 (5)
Feelings of worthlessness	22 (13)	10 (6)	8 (5)	83	12 (7)	2 (1)	14	10 (6)
Feeling lonely (intrapersonal)	20 (12)	5 (3)	3 (2)	67	15 (9)	7 (4)	44	10 (6)
Feeling of no longer being important to others	18 (11)	5 (3)	5 (3)	100	13 (8)	3 (2)	25	8 (5)
Feeling tired of life	17 (10)	5 (3)	5 (3)	100	12 (7)	4 (2)	29	9 (5)
Not satisfied with own self	12 (7)	5 (3)	5 (3)	100	7 (4)	2 (1)	25	7 (4)
Feelings of guilt	12 (7)	2 (1)	2 (1)	100	10 (6)	3 (2)	33	5 (3)
Experienced little success in life	10 (6)	2 (1)	0 (0)	0	8 (5)	2 (1)	20	2 (1)
Lived a life with little purpose	8 (5)	0 (0)	0 (0)	0	8 (5)	2 (2)	40	3 (2)
***Domain IV: Environment***								
Relatives consider your suffering too severe	33 (19)	10 (6)	9 (5)	83	23 (13)	7 (4)	31	16 (9)
Practical loneliness (no one present for you)	15 (9)	8 (5)	8 (5)	100	7 (4)	4 (2)	50	12 (7)
Insufficient availability of care	12 (7)	2 (1)	0 (0)	0	10 (6)	8 (5)	83	8 (5)
Unsatisfactory social contacts	8 (5)	2 (1)	2 (1)	100	6 (4)	1 (1)	25	3 (2)
Insufficient support (family, relatives)	5 (3)	0 (0)	0 (0)	0	5 (3)	2 (1)	33	2 (1)
Shame	2 (1)	0 (0)	0 (0)	0	2 (1)	2 (1)	100	2 (1)
***Domain V: Nature and prognosis of disease***								
Fear of future suffering	40 (24)	15 (9)	14 (8)	89	25 (15)	3 (2)	13	17 (10)
Fear of future failing strength to bear suffering	25 (15)	8 (5)	8 (5)	100	17 (10)	2 (1)	10	10 (6)

Rounded percentages and absolute numbers; 0 to 3 missing observations per symptom. Impaired working capacity applied for 10 persons.

Scoring: 1-not at all; 2-slightly; 3-moderately; 4-seriously; 5-very seriously, hardly can be worse.

Symptom present: intensity scores 2–5.

Low intensity: scores 1–3; High intensity: scores 4–5; Bearable suffering: scores 1–3; Unbearable suffering: scores 4–5.

*: percentage of interviewed patients.

n: number of patients.

### Intensity of symptoms in relationship to unbearability

Analysis of the relationship between intensity of symptoms and consequent unbearability for the most prevalent unbearable symptoms (25 symptoms were unbearable in at least 15%) demonstrated that high intensity of symptoms most frequently resulted in unbearable suffering for pain, loss of control over one’s life, fear of future suffering, not being able to do important things and not sleeping well (86-92%). Low intensity of symptoms most frequently resulted in unbearability for loss of control over one’s life (80%), vomiting (73%), not being able to do important things (52%), not sleeping well (43%) and loss of appetite (39%).

### Intensity of symptoms and unbearable suffering longitudinally

In 33 patients the SOS-V was administered at least two times. The pre-final interview on average was 123 days before death (SD 47 days). Prevalence of high symptom intensity significantly increased in the period between pre-final and final interview for general discomfort, being bedridden and help needed with self care (Table 4). Prevalence of unbearable suffering significantly increased for weakness.

**Table 4:** Development of intensity and unbearability of symptoms between the pre-final and final interview in patients with at least two interviews (n = 33)*

	**Symptom in high intensity†**			**Symptom unbearable†**		
	**Pre-final interview % (n)**	**Final interview % (n)**	**Difference pre-final and final interview % (95% CI) ‡**	**Pre-final interview % (n)**	**Final interview % (n)**	**Difference pre-final and final interview % (95% CI) ‡**
***Domain I: Medical symptoms***						
Weakness	36 (12)	55(18)	−18 (−29 to 3)	33 (11)	64(21)	**−30 (−41 to −6)**
General discomfort	9 (3)	27(9)	**−18 (−19 to −1)**	25 (8)	42(14)	- 16 (−33 to 9)
Tiredness	39 (13)	55(18)	−15 (−29 to 7)	42 (14)	42(14)	0 (−16 to 16)
Loss of appetite	27 (9)	18(6)	9 (−11 to 23)	27 (9)	36(12)	−9 (−26 to 13)
Pain	24 (8)	21(7)	3 (−11 to 14)	24 (8)	27(9)	−3 (−20 to 16)
Not sleeping well	12 (4)	6(2)	6 (−10 to 17)	9 (3)	24(8)	−15 (−26 to 5)
Changed appearance	30 (10)	42(14)	−12 (−29 to 11)	30 (10)	24(8)	6 (−12 to20)
Shortness of breath	6 (2)	21(7)	−15 (−15 to 0)	12 (4)	21(7)	−9 (−23 to 11)
Vomiting	3 (1)	12(4)	−9 (−9 to 4)	9 (3)	18(6)	−9 (−15 to 7)
Impaired co-ordination	12 (4)	12(4)	0 (−14 to 14)	24 (8)	18(6)	6 (−16 to 25)
Loss of concentration	21 (7)	9(3)	12 (−5 to 18)	16 (5)	18(6)	−3 (−18 to 14)
Memory loss	15 (5)	9(3)	6 (−7 to 12)	19 (6)	18(6)	0 (−11 to 11)
Incomprehensible speech	3 (1)	12(4)	−9 (−15 to 7)	9 (3)	9(3)	0 (−11 to 11)
***Domain II: Loss of function***						
Impaired routine daily activities	49 (16)	67(22)	−18 (−35 to 7)	55 (18)	61(20)	−6 (−27 to 18)
Impaired leisure activities	49 (16)	64(21)	−15 (−26 to 5)	42 (14)	52(17)	−9 (−15 to 6)
Help needed with housekeeping	39 (13)	55(18)	−15 (−29 to 7)	42 (14)	39(13)	3 (−16 to 10)
Bedridden	12 (4)	36(12)	**−24 (−30 to −5)**	18 (6)	36(12)	−18 (−24 to 1)
Help needed with self-care	6 (2)	27(9)	**−21 (−27 to −1)**	15 (5)	30(10)	−15 (−25 to 5)
***Domain III: Personal aspects***						
Not able to do important things	39 (13)	31(10)	9 (−11 to 24)	39 (13)	55(18)	−15 (−26 to 5)
Feeling dependant on others	33 (11)	55(18)	−21 (−32 to 1)	39 (13)	52(17)	−11 (−23 to 8)
Trouble accepting situation	21 (7)	36(12)	−15 (−26 to 5)	27 (9)	39(13)	−12 (−26 to 9)
Loss of control over own life	9 (3)	21(7)	−12 (−23 to 7)	16 (5)	30(10)	−13 (−23 to 8)
Negative thoughts or worrying	12 (4)	16(5)	−3 (−14 to 11)	18 (6)	18(6)	0 (−17 to 17)
***Domain IV: Environment***						
Relatives: suffering to severe	9 (3)	9(3)	0**	12 (4)	15(5)	−3 (−9 to 7)
***Domain V: Nature and prognosis of disease***						
Fear of future suffering	9 (3)	12(4)	−3 (−14 to 11)	13 (4)	12(4)	0 (−11 to 11)
Overall unbearable suffering				13 (4)	33(11)	−20 (−27 to 1)

* Selection of most frequent unbearable symptoms: symptoms which were unbearable (score 4 or 5) in at least 15% (9 patients) in the last interview for all patients with at least one interview; between 0–2 missing observations per symptom; three missing observations for overall unbearable suffering.

† High intensity: score 4 or 5 for intensity according to SOS-V; unbearable: score 4 or 5 for unbearability according to SOS-V.

‡ 95% confidence intervals calculated taking into account that results for pre-final and final interview are paired per patient; significant outcomes (0 outside of confidence interval) presented in bold.

** calculation of 95% CI not possible because scores are identical between pre-final and final interview for each patient.

### The nature of suffering: analysis of the qualitative data

Examples of qualitative answers of patients are provided in Table 5. The process of analysis of the qualitative data is demonstrated in Table 6. Overall unbearable suffering occurred in 28 percent (n = 16) of patients interviewed with the SOS-V. No significant difference in age, gender and cancer type occurred for present or absent overall unbearable suffering. The qualitative answers of the patients indicated physical suffering to be responsible for unbearable suffering with a mean of 4,0 in patients with overall unbearable suffering versus 2,1 in patients with overall bearable suffering (Table 7). Loss of meaning was present with a mean of 3,6 in patients with overall unbearable suffering, versus 2,0 in patients without overall unbearable suffering, while for loss of autonomy the mean numbers were 3,1 versus 2,2. All of these differences were statistically significant. Fear of future suffering and worrying were less prevalent overall, yet also went along with a mean higher number in patients with overall unbearable suffering. A remarkable repetitive explanation of unbearability of loss of appetite was the unwanted consequence of further loss of strength, related to not being able to eat.

**Table 5:** Personal descriptions of unbearable suffering by patients with high scores (score 4 or 5) on the SOS-V in the ultimate interview

	
General discomfort:	You just feel miserable, it feels like a sort of flue, you don’t fancy doing anything, you only want to lie in bed
Tired:	I hardly can do anything, it is nearly impossible to explain it in words, I never imagined a person could be so tired
Weakness:	My right leg is to weakened, it is not safe to stand and so for weeks I lie in my bed all the time and only come out for the latrine chair
Not sleeping well:	There is the noise at night on the streets, ambulances passing by, and this screaming neighbor woman, who keeps the whole neighborhood awake
Pain:	I have pain all day, it occupies my mind, there is little distraction
Loss of appetite:	I can hardly any more enjoy the taste of food, I long for the flavor of a fine stew
Thirst:	My mouth is dry, I need to drink, but this makes me nauseous
Smelling unpleasant:	I hate this urine smell
Changed appearance:	I used to be vain, now I have become so thin, I find it ugly looking at my neck
Impaired mental clarity:	I think about something, and then it’s gone, I find that stupid, for instance when I am one my way to fetch something
Concentration loss:	My mind loses its way, I find this unpleasant
Memory loss:	I used to remember all by mind, because I am illiterate
Feel tense:	I try to control it, for the children
Feel depressed:	This depressed mood in itself is unpleasant; at the same time it grows, because my daughter is doing less well
Feel anxious:	I feel afraid for what will come and worry about how things will go on for my wife
Shortness of breath:	Even with only slight activity I have a sort of hyperventilation, which makes me anxious
Coughing:	When in public, than this phlegm comes out in my handkerchief, it’s very annoying
Obstruction to swallow food:	I need to feed myself to prevent becoming even more weakened, but the passage of food is deranged
Obstruction to swallow fluid:	I swallow, but it doesn’t pass, it makes me retch
Nausea	I am nauseous continuously, I feel completely fed up with it
Vomiting:	When I sit at the table, it suddenly comes up and I need to run for the bathroom
Constipation:	It hurts and is strenuous
Diarrhea:	You are dining out in a hotel and then you continuously need to go to the toilet
Intestinal cramps:	These cramps are painful, it is very unpleasant
Incontinence of urine:	X (no high scores in ultimate interview)
Incontinence of feces:	It is filthy
Hiccups:	It comes sudden and unexpected, it makes me feel uncivilized and ashamed
Pressure ulcers:	It is annoying
Itch:	You keep on scratching
Skin metastasis:	In my neck, after radiotherapy it turned yellow, with an unpleasant look, it smells
Paralyzed limbs:	I can’t do anything, my left leg is paralyzed and my arm is forceless
Impaired coordination:	These unpredictable cramps and shaking of an arm or a leg, I cannot stop it
Incomprehensible speech:	I cannot communicate by telephone, people don’t understand what I am saying
Impaired comprehension of speech:	It makes me feel stupid
Dizziness:	Sometimes it is frightening, one time it happened when my alarm-button was out of reach and it took a whole long time before anybody entered and took notice
Impaired sight:	It annoys me so much, it is caused by the medication
Impaired hearing:	If some people talk at the same time I cannot differentiate what is being said
Impaired working capacity:	I am already counted out in society
Impaired in routine daily activities:	I am not the crying type, but this week I suddenly started crying
Impaired leisure activities:	I used to go out, make bus trips, I miss it, but absolutely can’t do it anymore. And I can’t any more receive people at my home, I am to exhausted
Need help with housekeeping:	Being young and not capable to function independently, it feels so unnatural
Need help with self-care:	I don’t want these young maidens of the home care service, it makes me feel ashamed
Pedridden:	More and more you are drawn to that bed, it makes you realize you are deteriorating
Restricted sexual functioning:	Widower: I feel rejected by my present partner, there is no intimacy, it is so cold
Not satisfied with own self:	I just left my wife, she had done nothing wrong, life than takes a course, leaving impossible to restore the situation
Lived a life with little purpose:	With my first wife everything was fine, the last years I miss love and tenderness
Experienced little success in life:	I would have liked to be at a somewhat higher level in society, for instance I would have liked to study, I would do it differently if I could do it again
Little happiness with family/close ones:	I would have preferred things to be different, I haven’t seen my children for 18 years
Trouble accepting situation:	I can no longer play Chopin, or make a drawing
Negative thoughts or worrying:	I would have liked to do things differently, at night it appears in my dreams
Feelings of guilt:	I feel guilt I wanted to divorce from my wife the other year, she is the one who makes I am still living now, she does everything for me
Feel worthless:	There is no more appreciation, people talk about you and not with you
Feel lonely:	One has cancer, it is not contagious, but people pass by less frequently
Feel hopeless:	This is not what I want
No longer feel the same person:	Is this the same body? Yet I have to manage with it, which causes me trouble
Feel tired of life:	I prefer it to be over as soon as possible, I used to be very active and independent and now I am totally passive and dependant
Feel dependant of others:	Ones individuality is lost, one has no more privacy
Feeling loss of control of life:	Tears in the eyes, gives no answer
Feeling a nuisance to others:	In relation to being so dependant I now easily tend to think “Oh, just leave it”
Feel unimportant to others:	My daughter, she is very sick and I can’t do anything for her
Impossible to do important things:	I used to daily visit my wife in a nursing-home, I can’t do it any longer
Not supported sufficiently by close ones:	The physical support is O.K., but there is emotional shortage
Lonely (important people absent):	I have one son, he does not visit me
Feelings of shame:	I have these outburst directed at my own person, which I find alarming, than this tic of my jaw appears and I wonder whether I can appear this way in church
Relatives consider your suffering severe:	It makes it difficult to start a conversation
Unsatisfactory contact with close ones:	They don’t keep stand up to their promises, for instance my daughter promises to visit me next week, I look forward to see her and then she shows up 1,5 years later
Insufficient availability of care:	I find the home care miserable
Fear of future suffering:	I feel short of breath, I am afraid to suffocate
Fear losing strength to bear the suffering:	To die, would it be painful?
Personal additions of missing aspects:	The hospice refused me last week, they considered me too good and advised a nursing home

**Table 6:** Symptom, description of what makes the symptom unbearable and rated category; examples

**Symptom**	**Description of unbearability by patient**	**Rated category**
Tired?	I hardly can do anything, it is nearly impossible to explain it in words. I never imagined a person could be so tired	Physical
Lonely (important people absent)?	I have one son, he does not visit me	Loss of meaning
Feel dependant on others?	I used to be very independent, and do all myself. Now I need to ask for everything, or wait.	Loss of autonomy
Hiccups?	It comes sudden and unexpected, it makes me feel uncivilized and ashamed	Loss of dignity
Feel to be a burden to others?	My husband, he needs to care for me continuously	Burden to others
Restricted sexual functioning?	It is gone, it is in pieces, not only for myself, but also for my wife	Loss of sexual role
Fear of future suffering?	I am in fear of suffering pain, that the pain will be unbearable	Fear of suffering
Nausea?	I feel panic. Am I going to vomit? Is it going to be difficult to breathe? Is it going to happen when I am eating?	Anxiety
Fear of future suffering?	I am frightened to suffocate	Death anxiety
Feel depressed?	This depressed mood in itself is unpleasant, at the same time it grows, because my daughter is doing less well	Depressive thoughts
Negative thoughts, worrying?	It haunts my mind all day: dissemination of cancer to my liver, 2 to 3 months to live. An operation? Other possibilities?	Worrying
Feel tensed?	To be able to be more relaxed would help me; now it makes me lose much energy	Feeling tensed
Hopelessness?	To take up a piece of paper , I can’t manage it, I cannot stand it	Hopelessness
Trouble accepting present situation?	The fact that it is as it is, to look it in the face. It is over, I am just waiting	Pointlessness

**Table 7:** Categories of suffering for patients who did or did not experience overall unbearable suffering (results from coding of open questions on why aspects of the SOS-V were unbearable)

	**Overall unbearable suffering (n = 16)**		**Overall bearable suffering (n = 41)**		
**Categories of suffering**	**Category present in patients % (n)**	**Mean number in which category is present per patient Mean (stdev)**	**Category present in patients % (n)**	**Mean number in which category is present per patient Mean (stdev)**	**p-value of t-test for means**
Physical suffering	100 (16)	4.0 (2.1)	61 (25)	2.1 (2.4)	0.008
Loss of meaning	88 (14)	3.6 (3.3)	63 (26)	2.0 (2.1)	0.027
Loss of autonomy	88 (14)	3.1 (2.2)	44 (18)	2.2 (1.6)	0.003
Loss of dignity	56 (9)	1.1 (1.5)	32 (13)	0,5 (0.9)	0.064
Experience to be a burden to others	56 (9)	0.7 (0.7)	17 (7)	0.2 (0.5)	0.025
Loss of sexual role	6 (1)	0.1 (0.3)	2 (1)	0.02 (0.2)	0.491
Fear of future suffering	44 (7)	0.8 (0.9)	5 (2)	0.04 (0.2)	0.009
Anxiety	31 (5)	0.6 (1.2)	7 (3)	0.1 (1.3)	0.089
Death anxiety	6 (1)	0.1 (0.5)	0 (0)	0.0 (0.0	0.333
Depressive thoughts	19 (3)	0.2 (0.4)	2 (1)	0.02 (0.2)	0.134
Worrying	44 (7)	0.5 (0.6)	5 (2)	0.1 (0.3)	0.020
Feeling tensed	13 (2)	0.1 (0.3)	5 (2)	0.05 (0.2)	0.417
Hopelessness	19 (3)	0.2 (0.4)	2 (1)	0.02 (0.2)	0.134
Pointlessness	13 (2)	0.4 (1.1)	2 (1)	0.4 (0.5)	0.299
Emotional symptoms	75 (12)	2.1 (2.6)	20 (8)	0.3 (0.8)	0.014

%: percentage of patients.

n: number of patients.

stdev: standard deviation.

Emotional symptoms of psychological distress: anxiety, death anxiety, depressive thoughts, worrying, feeling tensed, hopelessness and pointlessness.

## Discussion

The present study in end-of-life cancer patients in the home setting and in the care of primary care physicians demonstrated that weakness was the most prevalent physical symptom (93%), which was unbearable in approximately half of the patients. Pain was present in 72% of patients and was unbearable in a quarter of patients. A large variety of symptoms was present. If symptoms were of high intensity than unbearability frequently occurred for pain, loss of control, fear of future suffering, not being able to do important things and not sleeping well (86-92%). If symptoms were of low intensity than unbearability frequently occurred for loss of control, vomiting, not being able to do important things, not sleeping well and loss of appetite (39-80%). The prevalence of symptom intensity increased significantly between on average four months before death and on average one month before death for general discomfort, being bedridden and help needed with self care; the prevalence of unbearability significantly increased for weakness. Intensity and unbearability of pain were unchanged in the longitudinal follow-up. Evaluation of the qualitative study outcomes demonstrated that physical suffering, loss of autonomy, loss of meaning, fear of future suffering, experiencing to be a burden to others and worrying were significantly more prevalent in patients with overall unbearable suffering. The combined emotional symptoms of psychological distress were higher in patients with overall unbearable suffering.

### Strengths and limitations

The study was realized despite relevant barriers to research of end-of-live cancer patients in primary care (low prevalence of the studied patients, geographical dispersed setting of patients, physicians and researchers, difficulty of recruitment of end-of-life cancer populations for research) [25]. The 51% recruitment proportion of requested patients is comparable to recruitment proportions in secondary care studies investigating end-of-life cancer patients [10].

The study has limitations. The small sample size inhibited significant statistical analysis of the data of the relationship between intensity of symptoms and unbearability. Unexpected rapid physical deterioration limited the number of interviews shortly before death. The study sample concerns a Western population of patients in a specific setting of care, which limits generalizability.

### Comparison to other studies

No quantitative studies of unbearable suffering were found. Two studies qualitatively investigated unbearable suffering in mixed diagnostic populations (also other diagnoses than cancer) and identified physical, psychosocial and existential themes which contributed to the suffering [38,39]. In end-of-life cancer patients in secondary care overall moderate to extreme suffering was reported present in a range of 25%-81% [11,40,41], with physical symptoms, psychological distress and existential concerns contributing to the suffering [11]. The prevalent physical symptoms in this study, such as weakness, tiredness, general discomfort, changed appearance, pain and loss of appetite, are also prevalent in secondary care studies [24].

### Interpretation

Palliative home care has to meet the challenge of reducing unbearable suffering in end-of-life cancer patients. Between countries substantial differences in organization of palliative home care exist [2]. Palliative home care is provided in various models, such as general practitioner delivered palliative care [4,42] and home-based hospice care [43,44]. The services provided within these models are not standard [45] and few studies have assessed the intensity of palliative home care delivery in the last months of a patient’s life [46]. Transitions from oncology care to palliative care may influence patient well-being [47-49]. Palliative care is an accomplished formal medical specialization in some countries [50,51], providing possibility of structural integration of specialist palliative care within various health care settings, and providing possibility of educational fellowship rotations for various medical health specialties. In the Netherlands palliative care is not a specialty. Psycho-oncologic interventions provided by mental health professionals have been tested in various cancer populations [35,52-57]. Yet, for bed-ridden end-of-life cancer patients at home the contributions of psycho-oncologic interventions hardly are found applied or tested.

In the Netherlands legalized EPAS, with the compulsory criterion of unbearable suffering, most frequently concerns end-of-life cancer patients in primary care. It therefore appears that unbearable suffering prevalently is experienced in end-of-life cancer patients cared for in primary care. However, in the absence of studies it must be acknowledged that it is unknown which part of unbearably suffering patients die as a consequence of EPAS, nor is it clear which are the decisive steps from unbearable suffering to EPAS. Some specific situations in primary palliative care may influence suffering. Patients referred back from oncology care to home care, after hearing that no more treatment remains to slow down progress of cancer, may be demoralized [31]. Preference of cancer patients to die at home may inhibit interventions which require the setting of secondary care. In primary care selection occurs of patients with an explicit request for EPAS as a consequence of referrals by secondary care physicians of patients with a request for EPAS to their primary care physician. The frequency of these informal referrals is unknown. Mechanisms of transference and counter-transference about coping with suffering may be more prominent in primary care as a consequence of strong patient-physician relationships [58-61]. Further dynamics of care alter once a decision is made to proceed in a process towards EPAS [20,62].

Our study demonstrates many physical and psycho-social symptoms in end-of-life cancer patients cared for in primary care. Weakness was the most prevalent unbearable symptom. End-of-life cancer patients, in the home setting more than in hospital, may be confronted with loss of social role and autonomous functioning due to weakness. Pain, a potent cause of suffering [63,64], was unbearable in a relevant proportion of patients. The qualitative evaluation of the study indicates that core qualities of suffering were significantly more prevalent in patients who experienced overall suffering to be unbearable.

The results of our study underscore the need of adequate symptom control combined with psycho-oncologic interventions. Adequate control of adverse physical symptoms [65] makes it easier to address patients’ concerns about their families, about their own psychological integrity, and about meaning in their lives [30]. Existential aspects of suffering are addressed by psycho-oncologic interventions directed at meaning [29,53,57] and dignity [35,54]. Other types of interventions address acceptance and reactive emotional states [55,56]. Strength provided by the patient-physician relationship is another quality which may provide recovery from suffering [29,59,66,67]. The present study also shows that high intensity of symptoms does not necessarily indicate suffering, while low intensity of symptoms indeed may be unbearable.

We conclude that end-of-life cancer patients in primary care must cope with physical symptoms, loss of meaning, the emotional impact of suffering and the poor prognosis. Primary care physicians, as part of education in palliative care, therefore should be trained in understanding and diagnosing the multiple dimensions of suffering. The use of a framework of domains of suffering provides structure in assessment of suffering. It is not enough to assess the suffering: when meeting patients and taking their history, clinicians should keep a mental log of the issues that can be improved [47].

Further studies, as to improve understanding and develop interventions, should address unbearable suffering in various settings of care and investigate which are decisive steps from unbearable suffering to EPAS.

### Ethical approval

The study was approved by the Medical Ethics Committee at the VU University Medical Center (METC VUmc No. 2002/79).

## Competing interest

The authors declare that they have no competing interest.

## Authors’ contributions

CR had the initial idea for this study and wrote the initial research proposal. AK, GW and BOP commented on and contributed to the final research proposal. CR and BOP analyzed the data. CR wrote the paper, which was critically read by all the authors. All contributors had access to all the data and can take responsibility for the integrity of the data and the accuracy of the data analysis. All authors read and approved the final manuscript.

## Pre-publication history

The pre-publication history for this paper can be accessed here:

http://www.biomedcentral.com/1471-2296/14/201/prepub

## Supplementary Material

Additional file 1 State-Of-Suffering-V. Click here for file
