# Supplementary material for: Symptoms, unbearability and the nature of suffering in terminal cancer patients dying at home: a prospective primary care study
Source: BMC Fam Pract. 2013 Dec 28;14:201. doi: 10.1186/1471-2296-14-201 (PMC3877870; doi:10.1186/1471-2296-14-201)
Supplement: Additional file 1 — State-Of-Suffering-V. [file 1471-2296-14-201-S1.docx]

**Additional file 1: STATE-OF-SUFFERING- V**

SCORING SCALE **Not Slightly Moderately Seriously Very seriously,**

**at all hardly can be worse**

1-SYMPTOM PRESENT? 1 2 3 4 5

2-SYMPTOM UNBEARABLE? 1 2 3 4** 5**

**DOMAIN 1: MEDICAL SYMPTOMS**

***General*** Symptom present? Is it unbearable?

1-General discomfort? (feel miserable/feel unwell) 1-2-3-4-5 1-2-3-4-5

2-Tired? 1-2-3-4-5 1-2-3-4-5

3-Weakened? 1-2-3-4-5 1-2-3-4-5

4-Not sleeping well? 1-2-3-4-5 1-2-3-4-5

5-Pain? 1-2-3-4-5 1-2-3-4-5

6-Loss of appetite? 1-2-3-4-5 1-2-3-4-5

7-Thirst? 1-2-3-4-5 1-2-3-4-5

8-Smell unpleasant? ` 1-2-3-4-5 1-2-3-4-5

9-Changed appearance? 1-2-3-4-5 1-2-3-4-5

***Psychological***

10-Difficult to think clear? 1-2-3-4-5 1-2-3-4-5

11-Difficult to concentrate? 1-2-3-4-5 1-2-3-4-5

12-Memory loss? 1-2-3-4-5 1-2-3-4-5

13-Feel tensed? 1-2-3-4-5 1-2-3-4-5

14-Feel depressed? 1-2-3-4-5 1-2-3-4-5

15-Feel anxious? 1-2-3-4-5 1-2-3-4-5

***Respiratory tract, cardial***

16-Shortness of breath? 1-2-3-4-5 1-2-3-4-5

17-Coughing? 1-2-3-4-5 1-2-3-4-5

***Gastro-intestinal, urinary tract***

18-Swallowing and oesophageal passage for food impaired? 1-2-3-4-5 1-2-3-4-5

19-Swalowing and oesophageal passage for fluids impaired? 1-2-3-4-5 1-2-3-4-5

20-Nausea? 1-2-3-4-5 1-2-3-4-5

21-Vomiting? 1-2-3-4-5 1-2-3-4-5

22-Constipation? 1-2-3-4-5 1-2-3-4-5

23-Diarrhoea? 1-2-3-4-5 1-2-3-4-5

24-Abdominal cramps? 1-2-3-4-5 1-2-3-4-5

25-Involuntary loss of urine? 1-2-3-4-5 1-2-3-4-5

26-Involuntary loss of stool? 1-2-3-4-5 1-2-3-4-5

27-Hiccups? 1-2-3-4-5 1-2-3-4-5

***Skin***

28-Pressure ulcers? 1-2-3-4-5 1-2-3-4-5

29-Itch? 1-2-3-4-5 1-2-3-4-5

30-Ulcers caused by skin metastasis? 1-2-3-4-5 1-2-3-4-5

***Neurological, locomotor system***

31-Paralyzed limbs? 1-2-3-4-5 1-2-3-4-5

32-Impaired coordination of movements? 1-2-3-4-5 1-2-3-4-5

33-Incomprehensible speech? 1-2-3-4-5 1-2-3-4-5

34-Impaired comprehension of speech? 1-2-3-4-5 1-2-3-4-5

35-Dizziness? 1-2-3-4-5 1-2-3-4-5

36-Impaired sight? 1-2-3-4-5 1-2-3-4-5

37-Impaired hearing? 1-2-3-4-5 1-2-3-4-5

**DOMAIN II-LOSS OF FUNCTION**

Patient instruction: some of the following questions may not be applicable for you; please mention if such occurs.

Interviewer: Mark “X” when not applicable.

Symptom present? Is it unbearable?

38-Impaired working capacity? X 1-2-3-4-5 1-2-3-4-5

39-Impaired performance of routine daily activities? 1-2-3-4-5 1-2-3-4-5

40-Impaired leisure activities? 1-2-3-4-5 1-2-3-4-5

41-Need help with housekeeping? 1-2-3-4-5 1-2-3-4-5

(shopping, cleaning the house)

42-Need help with self care? 1-2-3-4-5 1-2-3-4-5

43-Bedridden? 1-2-3-4-5 1-2-3-4-5

44-Restricted sexual functioning? X 1-2-3-4-5 1-2-3-4-5

**DOMAIN III-PERSONAL ASPECTS**

Symptom present? Is it unbearable?

***Self esteem***

45-Not satisfied with your own self? 1-2-3-4-5 1-2-3-4-5

(with whom you are as a person)

46-Lived a life with little purpose? 1-2-3-4-5 1-2-3-4-5

47-Experienced little success in life? 1-2-3-4-5 1-2-3-4-5

48-Experienced little happiness with family/friends? 1-2-3-4-5 1-2-3-4-5

49-Trouble accepting the present situation? 1-2-3-4-5 1-2-3-4-5

50-Negative thoughts, worrying? 1-2-3-4-5 1-2-3-4-5

51-Feelings of guilt? 1-2-3-4-5 1-2-3-4-5

52-Feelings of worthlessness? 1-2-3-4-5 1-2-3-4-5

53-Feeling lonely? (intrapersonal) 1-2-3-4-5 1-2-3-4-5

54-Hopelessness? 1-2-3-4-5 1-2-3-4-5

55-Feeling not any longer being the same person? 1-2-3-4-5 1-2-3-4-5

56-Feeling tired of life? 1-2-3-4-5 1-2-3-4-5

***Loss of autonomy***

57-Feeling dependant on others? 1-2-3-4-5 1-2-3-4-5

58-Feeling loss of control over your life? 1-2-3-4-5 1-2-3-4-5

59-Feeling to be a burden to others? 1-2-3-4-5 1-2-3-4-5

***Future perspective***

60-Feeling not to be of importance to others in the

remaining time? 1-2-3-4-5 1-2-3-4-5

61-Feeling not to be able to do things you consider important

in the remaining time? 1-2-3-4-5 1-2-3-4-5

**DOMAIN IV: ASPECTS OF ENVIROMENT (SOCIAL, CARE),**

***Relationship with family and friends***

62-Experience insufficient support by family/relatives? 1-2-3-4-5 1-2-3-4-5

63-Feel lonely because the most important ones in your life

are not there for you? 1-2-3-4-5 1-2-3-4-5

64-Shame? 1-2-3-4-5 1-2-3-4-5

65-Do your relatives/friends consider your suffering too

severe? 1-2-3-4-5 1-2-3-4-5

***Communication***

66-Unsatisfactory relationships with relatives /friends? 1-2-3-4-5 1-2-3-4-5

***Aspects of* care**

67-Insufficient availability of care? 1-2-3-4-5 1-2-3-4-5

**DOMAIN IV: *Nature and prognosis of disease***

Symptom present? Is it unbearable?

68-Fear of future suffering? 1-2-3-4-5 1-2-3-4-5

69-Fear of not any longer having the strength to

bear the suffering? 1-2-3-4-5 1-2-3-4-5

MISSING ASPECTS

Instruct patient: are there important aspects of suffering which have not been mentioned? Which?

Symptom present? Is it unbearable?

70-………………………………………… 1-2-3-4-5 1-2-3-4-5

71-……………………………………….. 1-2-3-4-5 1-2-3-4-5

**TOTAL SCORE FOR SUFFERING**

Instruction of interviewer to patient: consider your situation as a whole for the next question

-How unbearable is your suffering overall? 1-2-3-4-5

**Score 4 or 5 for unbearability: additional open qualitative questions about the nature of the suffering

Additional instructions:

-the X for impaired working capacity is to identify the working population

-question about help needed with housekeeping: address the situation of the interviewed person and not the situation of the system in which the interviewed person functions (for instance a partner who does the housekeeping)
